# Targeting Platelets for the Treatment of Cancer

**DOI:** 10.3390/cancers9070094

**Published:** 2017-07-22

**Authors:** Omar Elaskalani, Michael C. Berndt, Marco Falasca, Pat Metharom

**Affiliations:** 1 Faculty of Health Sciences, Curtin University, Perth 6100, Australia; omar.elaskalani@postgrad.curtin.edu.au (O.E.); m.berndt@curtin.edu.au (M.C.B.); marco.falasca@curtin.edu.au (M.F.); 2 Curtin Health Innovation Research Institute (CHIRI), Curtin University, Perth 6100, Australia; 3 School of Biomedical Sciences, Curtin University, Perth 6100, Australia

**Keywords:** cancer, cancer therapy, platelet, antiplatelet

## Abstract

The majority of cancer-associated mortality results from the ability of tumour cells to metastasise leading to multifunctional organ failure and death. Disseminated tumour cells in the blood circulation are faced with major challenges such as rheological shear stresses and cell-mediated cytotoxicity mediated by natural killer cells. Nevertheless, circulating tumour cells with metastatic ability appear equipped to exploit host cells to aid their survival. Despite the long interest in targeting tumour-associated host cells such as platelets for cancer treatment, the clinical benefit of this strategy is still under question. In this review, we provide a summary of the latest mechanistic and clinical evidence to evaluate the validity of targeting platelets in cancer.

## 1. Introduction

Metastasis and acquired chemotherapy resistance are major obstacles in the treatment of patients with cancer. Recent seminal studies by Erpenbek and Schon [1] and Labelle et al. [2] have rekindled renewed interest into the role of platelets in cancer survival and metastasis. Using a mouse pulmonary melanoma metastasis model, Erpenbeck and Schon [1] demonstrated a critical role for platelets in metastasis since metastasis was totally blocked by platelet depletion. Potential adhesion receptors involved in mediating the augmentation of metastasis included the platelet-specific integrin, αIIbβ3, and P-selectin. The seminal paper by Labelle M et al. [2] identified a key role for platelets in mediating epithelial-mesenchymal transition (EMT) in circulating cancer cells.

Apart from their role as a key regulator in haemostasis [3], platelets can also mediate host immune and inflammatory responses [4,5]. A large number of experimental and clinical data underpin a pro-metastatic role of platelets in cancer [2,6,7,8]. It is well accepted that some tumour cells can stimulate platelets [9,10]. Once activated, platelets release an array of biologically active molecules which can modulate tumour growth and metastasis [11,12,13]. Within the blood compartment, tumour cells can form aggregates with platelets and thus avoid natural killer cell mediated cytotoxicity [14,15,16]. Therefore, tumour cell adhesion of platelets and their activation is a crucial step for tumour cell survival within the blood circulation [17,18].

To facilitate adhesion to platelets, some cancer cells can upregulate aberrant surface proteins. For example, lung cancer cells express P-selectin glycoprotein ligand 1 (PSGL-1), a protein commonly found on white blood cells, that binds with high affinity to P-selectin on the surface of activated platelets [19]. Selected cancer cells can also express podoplanin, a protein which can elicit platelet activation and aggregation through interaction with the C-type lectin-like receptor-2 (CLEC-2) on platelets [20]. In addition to direct physical interaction with tumour cells, platelets support cancer progression by regulating tumour angiogenesis through a variety of secreted biological factors, e.g., vascular endothelial growth factor (VEGF), dopamine, serotonin and endostatin [21,22,23,24,25].

Additionally, platelets can modulate the behaviour of tumour cells. Platelets are the major storage site for transforming growth factor beta 1 (TGFβ1) within the blood circulation, which is released from α-granules upon activation [26]. Platelet-derived TGFβ1 can promote an epithelial-mesenchymal transition in cancer cells, an essential step in cancer invasion and metastasis [2]. While the majority of research so far has focused on the role of platelets in cancer metastasis and angiogenesis, recent data suggest an expanding role of platelets in tumour development, particularly their potential contribution to chemoresistance and cancer growth [27,28]. In this regard, lyso-phosphatidic acid (LPA) and platelet factor 4 (PF4) are also released from activated platelets and been shown to positively enhance tumour growth [29,30].

The study of platelet function in cancer patients has many challenges. In addition to disturbances of blood physiology due to cancer and the patient’s clinical state [31,32,33], many chemotherapeutic agents themselves can directly affect the behaviour of platelets [34,35]. Moreover, some cancers and certain therapeutic regimens can lower the number of blood cells and platelets in the circulation, resulting in adverse side-effects such as anaemia and bleeding issues that raise ethical and practical concerns in acquiring samples for research purposes. The majority of the literature on platelet-tumour crosstalk has used platelets from healthy volunteers or mouse tumour models for experimental research which may not accurately mirror the disease in human. Although many aspects of the interactions can be delineated via these approaches, whether these models are informative for clinical trial development is still under investigation. This review highlights current literature in regard to the benefit of targeting platelets in cancer therapy.

## 2. The Role of Platelets in Cancer Metastasis

Cancer metastasis requires changes in cancer cells that lead to a more aggressive phenotype, characterised by an elongated shape, high motility and invasive capacity [36,37,38,39]. In fact, the majority of cancer-associated mortality results from the ability of tumour cells to invade secondary sites, leading to multifunctional organ failure and death. Inside the primary tumour, cancer cells utilise autocrine and paracrine growth signals provided by other tumour cells and stroma cells [40,41]. Furthermore, within the blood circulation, platelet-cloaked tumour cells can bypass natural killer cell-mediated cytotoxicity [15].

Platelets are a major storage site for TGFβ1 [26]. Activated platelets can supply sufficient TGFβ1 to enable successful metastasis of tumour cells. Tumour cells primed with platelets in vitro showed increased metastasis after injection into mice [2,8]. Mechanistically, platelet-derived TGFβ1 acts via the p-Smad pathway to induce a phenotypic conversion in cancer cells, from epithelial to mesenchymal-like cells, capable of invading extracellular matrices, migrating and surviving in the blood circulation [42,43]. Epithelial-mesenchymal transition (EMT) is characterised by upregulation of mesenchymal-like proteins such as N-cadherin, Slug, Snail, vimentin and either downregulation, translocation or loss of function of epithelial-like proteins such as E-cadherin and claudin-1 (reviewed by Xu et al.) [44]. Soluble platelet-derived factors (mainly TGFβ1) and direct physical contact with tumour cells (activating NF-κB pathway) work synergistically to induce EMT and subsequent migration and metastasis [2,45].

In addition to their pro-EMT role, Labelle et al. [46] provided evidence for the prominent role of platelets in early metastatic niche formation. Platelet-derived, but not tumour-derived signals, as well as platelet aggregates around tumour cells, are essential for granulocyte recruitment to the early metastatic niche [46]. Platelet-derived chemoattractants such as C-X-C motif ligands (CXCL5/7) specifically induce deployment of granulocytes and not monocytes, lymphocytes or NK cells to the early metastatic niches [46]. Similarly, Orellana et al., recently demonstrated the chemotactic effect of platelets on ovarian cancer cells, with the subsequent phenotypic change favouring a mesenchymal phenotype [47]. Therefore, platelets not only provide survival signals for tumour cells but also recruit host cells to the disseminated tumour foci.

**Figure 1:**
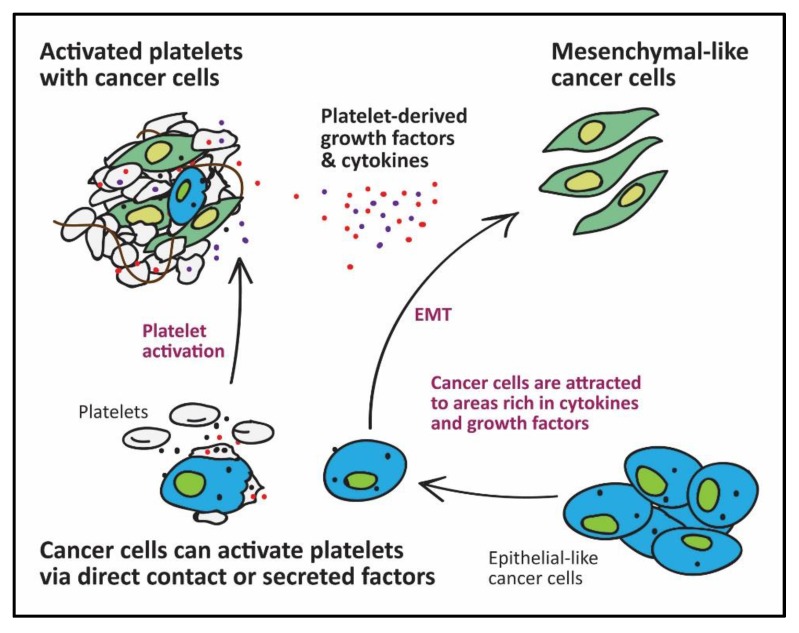
Platelets in Metastasis: Cancer cells can activate platelets. Activated platelets secrete growth factors and chemokines to attract other cancer cells to areas rich in survival factors. Platelet TGFβ1 induces EMT in cancer cells, which are characterised by an elongated shape and improved metastatic ability.

**Figure 2:**
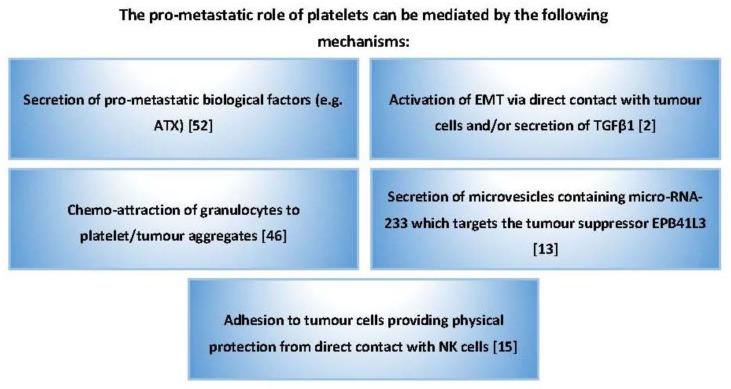
Summary of the pro-metastatic properties of platelets in cancer. Platelets promote cancer progression by releasing an array of pro-metastatic biological factors and by shielding cancer cells from NK-mediated cytotoxicity.

Besides TGFβ1 and chemo-attractants, activated platelets also secrete autotaxin (ATX); an enzyme with phospholipase D activity that generates LPA from lysophosphatidylcholine and contributes to cancer progression by promoting cancer proliferation, angiogenesis and metastasis [48,49,50,51,52]. Moreover, platelet-derived microvesicles contain micro-RNA-233, which can enhance lung cancer cell invasion by directly targeting the tumour suppressor EPB41L3 [13]. Platelets appear to be integral to the cancer metastasis processes either by directly interacting with cancer cells, attracting other host cells to the hetero-aggregate site, or even by attracting tumour cells to a location rich with survival factors (Figure 1 and Figure 2).

## 3. The Role of Platelets in Tumour Angiogenesis

The tumour microenvironment plays a major role in cancer progression. In 1971, Folkman [53] described the requirement of endothelial cell-mediated neovascularization for tumour growth and survival. Tumour cells release growth factors that stimulate angiogenesis, defined by the regeneration of endothelial cells to form new blood vessels from pre-existing ones. Newly formed blood vessels supply the dividing tumour cells with blood and oxygen. Thus, angiogenesis is a fundamental process for tumour growth and survival, which is governed by a balance between pro- and anti-angiogenic factors [53].

Platelets contain a diverse range of biological molecules that can regulate angiogenesis [25], for example, dopamine and serotonin, neurotransmitters synthesised in the central nervous system with a well-established role in mediating numerous neurological and psychological processes. Drugs that target their synthesis are clinically available for controlling several pathological conditions [54,55]. In the blood circulation, dopamine and serotonin are predominantly stored in the dense granules of platelets and released upon platelet activation [21,56]. Dopamine plays a significant role in inhibiting angiogenesis. Intraperitoneal injection of dopamine was able to block angiogenesis and tumour growth in an in vivo mouse model where it inhibited VEGF/vascular permeability factor (VPA)-mediated proliferation and migration of human umbilical vein endothelial cells (HUVEC) [22]. Interestingly, daily dopamine use blocks stress-mediated tumour growth and angiogenesis in vivo [57]. The activity of dopamine was attributed to its action on the dopamine-2-receptor on endothelial cells, resulting in impaired VEGF-mediated phosphorylation of VEGF receptor-2 (VEGF-R2) [22,58,59]. It has been hypothesised that dopamine induces endocytosis of VEGF-R2, resulting in fewer receptors available for VEGF binding. Furthermore, pretreatment of HUVEC with dopamine hampers the disruptive effect of VEGF on zonula occuldens (ZO-1) protein, a tight junction protein that preserves endothelial cell-cell adhesion [22,60].

In addition to its role in promoting cancer cell proliferation [61,62,63], serotonin can activate angiogenesis by promoting proliferation of endothelial cells through the activation of several signalling kinases; for example, Src, PI3K, AKT, ERK, and mTOR. Interestingly, the downstream signalling pathway mediated by serotonin is shared with VEGF [23].

VEGF is a chemotactic vascular permeability factor stored in α-granules and released from activated platelets. It promotes angiogenesis through VEGF-R2 on endothelial cells [64,65]. Tumour VEGF stimulates the release of von Willebrand factor from endothelial cells. Together with increased permeability of endothelial cells and exposure of subendothelial proteins like collagen, platelets are attracted, activated and adherent to the tumour-activated endothelial cells (hypothesis by Pinedo et al. [66]). VEGF action is transduced via tyrosine kinases and ultimately leads to endothelial cell proliferation and formation of new blood vessels to boost tumour growth and survival [67]. Activated platelets can secrete either pro- or anti-angiogenic factors, depending on the nature of the stimulant [24,68]. In addition to their ability to release angiogenic factors upon activation, platelets can also sequester VEGF, as evidenced by the preferential accumulation of VEGF in platelets compared to plasma or other cells after subcutaneous injection of radiolabeled VEGF into mice [69,70]. Wu et al., have recently demonstrated that non-small cell lung cancer (A549)-activated platelet releasate can stimulate angiogenesis even in the presence of VEGF neutralising antibody [71]. Therefore, platelet-derived VEGF contributes to tumour angiogenesis but is not essential as activated platelets can also release microvesicles and exosomes to induce expression of angiogenic factors in cancer cells [11].

Platelet-derived angiogenic factors are also relevant as useful prognostic markers. For example, platelets isolated from breast cancer patients contain a higher level of VEGF and angiopoietin 1 (Ang-1), while platelets from prostate cancer patients show a greater level of VEGF but not Ang-1 [72]. Similarly, platelets isolated from breast cancer patients display a higher degree of pro-angiogenic and metastatic growth factors; for example, TGFβ1, VEGF, and platelet-derived growth factor (PDGF), compared to a control group [73]. Platelet VEGF levels correlate with angiogenesis and staging in non-Hodgkin lymphoma [74]. Peterson et al., have evaluated the normal physiological ranges of angiogenesis regulators relevant to monitoring cancer prognosis and response to anti-angiogenesis therapy [75] (see Table 1).

## 4. The Role of Platelets in Tumour Growth

The ability of cancer cells to trigger secretion of growth factors from platelets has attracted a widespread interest of a potential role of platelets in cancer cell growth and proliferation. Platelets are a rich source of biologically active molecules, and there is evidence that different platelet agonists can elicit different patterns of release from platelets [24,76,77]. Accordingly, various cancer cells could potentially induce different patterns of platelet secretion. Accumulating evidence has established a pro-metastatic role of platelets in cancer. However, the impact of platelets on cancer cell proliferation is controversial, confounded by variable findings in different cancer cell types.

More than 30 years ago, Ibele et al. [78] suggested that platelets may play a role in host defence against malignant tumours. Moreover, monocytes in the presence of platelets showed higher tumour killing capacity compared to monocytes alone. Surprisingly, aspirin decreased the cytotoxic effect of platelets on tumour cells, highlighting a potential toxic effect of platelet arachidonate metabolites on cancer cells [78,79]. Similarly, unstimulated and thrombin-activated platelets showed tumoricidal activity with the chronic myelogenous leukaemia cell line, K562, an effect that was completely blocked by esterase inhibitors in unstimulated platelets but not in thrombin-activated platelets. In contrast, some cancer cell lines were resistant to the cytotoxicity of platelets. The authors proposed an explanation for their findings in concordance with emerging studies that established a pro-metastatic role of platelets; the cytotoxic effect of platelets being mainly relevant to sensitive cancer cells while resistant cells were not affected. The formation of hetero-aggregates of platelets and resistant tumour cells on the endothelial surface would trigger the release of cytotoxic factors from platelets, causing injury to the endothelium, thus creating pores for resistant cancer cells to penetrate and metastasize [80]. Therefore, the cytotoxic effect of platelets may promote the survival of aggressive cancer cells.

Platelets contain immune defence factors such as pro-apoptotic members of the tumour necrosis factor (TNF) family, including tumour related apoptosis inducing ligand (TRAIL), TNFα, CD154 and Fas ligand (Fas-L) [81]. Fas-L is expressed and released by platelets after activation with ADP or thrombin. The interaction between Fas-L/Fas receptor leads to activation of the caspase-mediated apoptosis pathway in tumour cells that express Fas-R, such as adult T-cell leukaemia (CEM) [82]. Using mouse cancer cell lines and platelets, Wang et al., also examined the impact of platelets on cancer cell proliferation. Although platelets did not activate apoptosis in various cancer cell lines, they decreased proliferation by inducing cell cycle arrest [83].

In contrast to the reports of tumoricidal activity mentioned above, platelets augment the proliferation of ovarian cancer cells in vitro and in vivo, independent of platelet adhesion to cancer cells, an action mediated mainly through TGFβ1 [84,85]. Additionally, a recent paper by Haemmerle et al. also highlights an important role of platelet’s protein, focal adhesion kinase (FAK), in mediating platelet infiltration and tumour growth in ovarian cancer mouse model [85]. Thrombocytosis, commonly referred to a platelet count >400–450,000 per millilitre of blood, is observed in approximately one-third of women who have been recently diagnosed with ovarian cancer [86,87,88]. In addition to thrombocytosis, reports of thrombophilia (a hypercoagulable state) and tumour-infiltrating platelets are closely associated with an advanced-stage disease, and a poor prognosis [86,89]. It has been proposed that the high platelet count is the result of tumour-derived plasma interleukin-6 (IL-6), which can mediate the synthesis of thrombopoietin (a hormone responsible for regulating platelet production) in the liver to stimulate platelet production [86].

Infiltration of platelets into solid tumours has also been demonstrated in colorectal cancer, hepatocellular carcinoma, breast cancer and gastric cancer, and their presence was associated with tumour growth in insulinoma and melanoma mouse models [90]. More recently, Pucci et al., have delineated the impact of PF4 on cancer progression; PF4 enhanced platelet production and accumulation at the tumour site, which accelerated lung adenocarcinogenesis in a genetically modified mouse model. Similarly, platelets exert a pro-proliferative effect on a panel of hepatocellular carcinoma cell lines by activating the MAPK pathway and decreasing apoptotic effectors. However, the nature of the growth factor(s) responsible for this effect was not defined by the authors [91].

In 1984, Tucker and colleagues demonstrated a role for platelet TGFβ1 in the proliferation of different cell lines (mouse embryo-derived cells (AKR-2B), rat kidney-derived cells (NRK), African green monkey kidney cells (BSC-1) and mink lung cells (CCL-64). Interestingly, platelet TGFβ1 showed different (stimulatory or inhibitory) effects on proliferation based on the experimental conditions (cells growing as unattached rounded cells in soft agar or as an adherent monolayer) [92]. Likewise, Roberts et al., demonstrated a bifunctional role of TGFβ1 on different human cancer cell lines. In their study, TGFβ1 significantly reduced colony formation with the lung cancer cell line (A549) and breast cancer cells (MCF-7), while it had no effect on colon cancer cells (HT-29). Also, TGFβ1 could either potentiate or antagonise the effect of other growth factors, such as platelet-derived growth factor (PDGF) and epidermal growth factor (EGF) [93].

Overall, the secretome of platelets contains a multitude of biologically active factors, with a net effect dependent on the interactions between platelet-derived factors, tumour-derived factors and tumour receptors. In addition, the role of platelets in tumour growth is highly dependent on tumour type; as different platelet stimuli have been suggested to trigger a different pattern of platelet release, cancer cell-induced platelet secretion may function in a similar way [24,96]. One of the possible reasons for the contradictory results of some of the early in vitro studies is the variable ratio of platelets to tumour cells, which may differ from the expected ratio in cancer patients. For example, thrombocytosis is often found in cancer patients before treatment (surgery, chemotherapy or radiotherapy) [97,98]. However, use of some chemotherapeutic agents is associated with low platelet count (Table 2). Therefore, the platelet to cancer cell ratio varies through the course of the disease and is highly affected by treatment. Nonetheless, studies that have utilised genetically modified mouse models indicate a positive influence of platelets on tumour growth (Table 3). Moreover, some of the platelet-derived biological factors (e.g., PDGF, TGFβ1, ATX) have been studied independently of platelets and showed an active role in cancer progression [44,48,50,99].

Table 3 summarises the current understanding of the role of platelets on tumour growth. Unlike numerous in vitro studies which suggest an anti-tumour effect of platelets, the pro-tumour role of platelets is well established in several mouse models [100].

## 5. The Role of Platelets in Chemotherapy Resistance

The ability of malignant tumours to grow despite chemotherapy is considered a significant contribution to treatment failure and low survival rates associated with highly resistant types of tumours, such as pancreatic cancer. Malignant tumours are usually made up of multiple populations of cancer cells which differ in their metastatic ability and response to chemotherapy. The more resistant cancer cells eventually dominate the tumour as the more sensitive cells are eradicated by chemotherapy. The principal mechanisms mediating chemotherapy resistance are enhanced proliferation of cancer cells through activation of the MAPK signalling pathway, activation of anti-apoptotic proteins, or phenotypic conversion in cancer cells through the epithelial-mesenchymal transition, all of which could potentially be affected by platelets. The platelet secretome is rich in growth factors and is used clinically to enhance tissue regeneration [103]. During the wound healing process, platelets display a pro-proliferative role through the secretion of various growth factors. Platelets thus possess the ability to counter the anti-proliferative effect of chemotherapeutic agents.

Currently, only a few studies have examined the contribution of platelets to chemotherapy resistance. In 2012, Balicka et al. demonstrated a role for platelets in paclitaxel and 5-fluorouracil resistance in colon (Caco-2) and ovarian (59 M) cancer cells [27]. Platelets and their releasate antagonised the cytotoxic effect of paclitaxel and 5-fluorouracil via several complementary mechanisms. First, by shifting the balance between anti-apoptotic and pro-apoptotic genes towards cell survival through upregulation of anti-apoptotic proteins such as NFκB1. Second, by blocking cell cycle arrest caused by the anticancer drugs. This occurred through upregulation of cyclins, the principal regulators of cell cycle progression. Third, platelets also enhanced the phosphorylation of DNA repair proteins, for example, Chk1, BRCA1, and Mre11. Moreover, platelets upregulated the MAPK signalling pathway, which is involved in cell growth, invasion, and migration [27]. Similarly, D’Alessandro et al. recently demonstrated the ability of platelet factors to hinder the cytotoxicity of the chemotherapy drugs, sorafenib and regorafenib, in hepatocellular carcinoma by increasing the phosphorylation of ERK, p38 and by inhibiting the induction of apoptosis Moreover, platelets also counteracted the efficiency of both drugs in halting cancer cell migration and invasion [104]. Clinically, chemotherapy resistance has been correlated with high platelet count [105].

Platelets also drive EMT in cancer cells with a subsequent increase in migration, invasion and metastasis [2]. The presence of platelets around breast cancer primary tumour cells was associated with EMT morphological features and chemotherapy resistance [106]. Independent of platelet activity, EMT has been shown to impart chemotherapy resistance in lung [107], pancreatic [108], breast [109] and ovarian cancers [110]. Zheng et al., demonstrated using a pancreatic adenocarcinoma mouse model with deleted mesenchymal transcriptional factors, Snail and Twist, that EMT inhibition did not prevent metastasis but contributed significantly to enhanced gemcitabine sensitivity [111]. Pancreatic cancer is a highly metastatic type of cancer, and is known to trigger platelet activation, aggregation, and secretion [31,112,113]. Platelets are considered the primary source of TGFβ1 in the blood circulation, which is a primary inducer of EMT [2]. Therefore, targeting the activity of platelets in cancer may not only diminish cancer metastasis but also suppress chemotherapy resistance. Chemotherapy in combination with antiplatelet therapy may thus represent a potential approach to overcome tumour chemoresistance.

## 6. The Effects of Cancer Cells on Platelets

Cancer cells can directly trigger platelet activation by releasing factors that act as agonists or by direct physical contact [20,114,115]. One of the best characterised mechanisms of tumour cell-induced platelet activation is through podoplanin/CLEC-2 interaction. Podoplanin (PDPN) is a transmembrane sialoglycoprotein highly expressed on metastatic cancer cells. It is also found on tumour-initiating cells and is associated with poor prognosis in lung adenocarcinoma [116]. PDPN can trigger platelet activation, aggregation and secretion through interaction with the CLEC-2 receptor on the surface of platelets. MS-1, an anti-PDPN antibody, which blocks PDPN/CLEC-2 interaction, significantly reduced tumour metastasis and tumour growth in vivo [117]. Direct contact with platelets, however, is not always required to trigger activation as ADP released from cancer cells can activate platelet P2Y_1_ and P2Y_12_ receptors [9,118,119], while an unknown factor released from prostate cancer cells can instigate activation of platelets through the FcƳRIIa receptor [115].

Moreover, cancer cells can activate platelets indirectly through the coagulation pathway. The procoagulant potential of different tumours is highly dependent on their expression of tissue factor (TF). TF mediates thrombin generation through activation of the extrinsic pathway of coagulation, which can directly activate platelets [120]. Tissue factor has also been found in tumour-derived microvesicles associated with enhanced venous thromboembolism in mice [121]. Cancer cells of different origin express varying levels of TF. For example, the pancreatic cancer cell line BXPC3 expresses a higher level of TF compared to the breast cancer cell line MCF7 [122]. Notably, pancreatic cancer is highly associated with venous thromboembolism, which can be related to TF either expressed or released by pancreatic cancer cells [31,121,123]. On the other hand, platelets can promote TF expression in cancer cells as shown with ovarian cancer cells co-cultured with platelets [47]. Ovarian cancer is also associated with a high risk of venous thromboembolism [124]. In addition to tissue factor, cancer cells can activate platelets indirectly through eliciting neutrophils to release neutrophil extracellular trap (NET). NETs are an extracellular mesh of DNA associated with histones, elastases and myeloperoxidase (MPO), previously known for their antimicrobial function [125]. Recent studies have shown the ability of cancer cells to prime and induce neutrophils to generate NETs which are associated with thrombus formation [126,127]. Furthermore, NETs can instigate platelet activation and aggregation [128,129,130].

**Figure 3:**
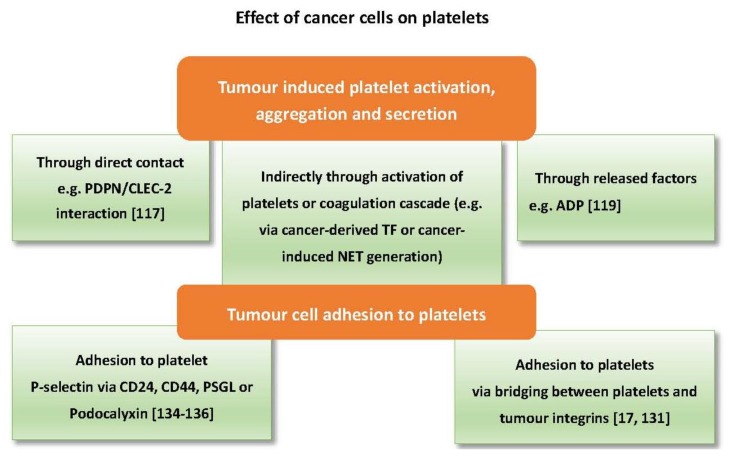
Summary of cancer cell-platelet interactions. Cancer cells can activate platelets through expression or release of platelet agonists (e.g., PDPN and ADP). Cancer cell-derived tissue factor (TF) can also indirectly activate platelets via the coagulation cascade and generation of thrombin. Cancer cells can also express ligands (e.g., CD24, PSGL, and integrins) that facilitate cancer cell-platelet adhesion.

As mentioned earlier, cancer cell adhesion to platelets is vital for successful metastasis, which can be mediated through surface proteins and predispose platelet activation. For example, interaction between integrins (transmembrane glycoproteins) expressed on platelets (e.g., αIIbβ3), and some types of tumour cells via ligands that are normally present in plasma such as fibrinogen and fibronectin can lead to activation of platelets. Integrins are involved in tumour-platelet adhesion and subsequent tumour arrest within the blood circulation [17,131]. In addition to integrins, tumour cells can adhere to platelets via the P-selectin ligands. PSGL-1 as acts as a P-selectin counter-receptor in non-small cell lung cancer cells, multiple myeloma cells, and prostate cancer cells [19,132,133]. Other P-selectin ligands, CD24 and CD44, are found on breast cancer cells and colon cancer cells, respectively [134,135]. In mice, engagement of P-selectin by PSGL-1 results in platelet activation and enhanced micro-aggregate formationwhile P-selectin null mice display inadequate thrombus formation [136]. Finally, podocalyxin is a membrane mucin protein expressed by testicular cancer cells that can also mediate platelet adhesion via P-selectin and integrins [137]. Figure 3 summarises the effects of cancer cells on platelets.

## 7. Challenges to Antiplatelet Therapeutic Approaches in Cancer

There is increasing evidence supporting an active role of non-cancer cells within the tumour microenvironment in cancer progression, thus introducing additional strategies in cancer therapy in which different classes of drugs could be combined to target different cell types that collectively would impede tumour growth and metastasis. Available preclinical data provide examples of this approach; for instance, in a mouse tumour model, a treatment combining low dose cyclophosphamide with the thrombin inhibitor, Dabigatran etixulate, reduced tumour growth and metastasis through potentially limiting tumour-platelet crosstalk [138]. In addition to its role in the direct activation of platelets, thrombin generates fibrin. Some cancer cells can also release thrombin [114].

**Figure 4:**
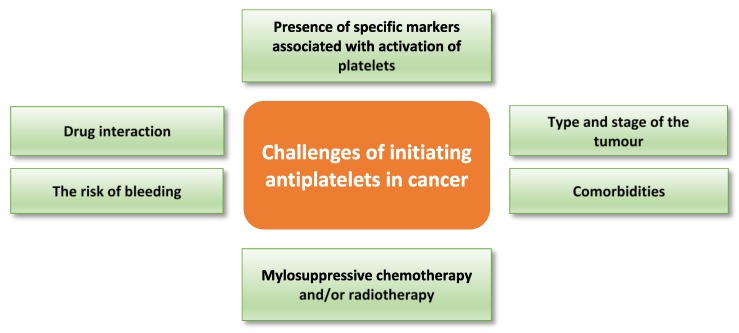
Potential challenges in targeting platelets during cancer therapy. Clinical implementation of antiplatelets in cancer may face several confounders which can be patient-related or therapy-related.

The use of antiplatelets in cancer therapy may be confounded due to declining platelet function as a consequence of disease progression, myelosuppressive chemotherapy and/or radiotherapy (reviewed by Liebman) [139]. For example, platelets obtained from thrombocytopenic cancer patients before platelet transfusion have shown impaired responses to thrombin, collagen-related peptide, and ADP as measured by αIIbβ3 activation and P-selectin translocation [140]. Thus, bleeding risk needs to be carefully evaluated, especially in cancer patients with comorbidities such as cardiovascular disease, before use of any antiplatelet drug. Although there is a well-established pro-metastatic role of platelets in cancer, the effect of platelets on cancer progression could vary based on type and stage of the tumour. Thus, the addition of antiplatelet treatment to cancer therapy should be individualised based on the clinical and experimental evaluation. Many factors must be considered before deciding to administer antiplatelets in cancer therapy; these include the risk of bleeding, comorbidity, chemotherapy and radiotherapy dose and duration, drug interaction, and type and stage of the tumour. Table 2 highlights the effect of a group of cytotoxic drugs on the platelet count. Platelet studies and presence of specific markers of platelet activation are among the experimental factors that should be assessed before administering antiplatelet drugs to cancer patients. Figure 4 highlights potential challenges with targeting platelets in cancer therapy.

## 8. Clinical and Preclinical Use of Antiplatelet Therapies in Cancer

### 8.1. Aspirin in Cancer

The impact of a common household drug, aspirin, on cancer progression has attracted considerable interest. Here, we present an overview of the anti-platelet and anti-metastatic efficacy of aspirin. Despite the encouraging results from preclinical models and the molecular rationale, the results obtained in human trials are less clear.

#### 8.1.1. Preclinical Studies

In 1962, Gasic et al. reported a reduction in metastasis of TA3 tumour cells in mice injected with *Vibrio* Cholera neuraminidase (VCN); a potent thrombocytopenic agent [141]. In a subsequent study by the same group, tumour cells that were able to aggregate platelets in vitro showed more lung metastasis compared to tumour cells devoid of this ability. Furthermore, platelet-deficient mice showed reduced lung metastasis from tumours that aggregated platelets in vitro. In contrast, tumour cells that did not aggregate platelets in vitro still formed metastases in thrombocytopenic mice although the number of metastatic foci was fewer. Interestingly, aspirin significantly decreased lung metastasis without affecting the size of the primary tumour [6].

In another study, pre-incubating platelets with aspirin inhibited murine sarcoma cells (mFS6)-induced platelet aggregation [142]. Similarly, Bradley et al., demonstrated a pro-aggregation effect of uterine carcinosarcoma (Colo 562) cells on human washed platelets. However, aspirin did not prevent platelet adhesion to tumour cells, platelet secretion or micro-aggregate formation [143]. Further, in an in vivo model, aspirin reduced lung metastasis of rat mammary carcinoma (Mtln3) but did not provide an additive effect when combined with the fibrinolytic agent, streptokinase, which itself caused a significant reduction in metastasis [144]. In contrast, a combination of aspirin and ATP102 (an ADPase) significantly decreased breast cancer and melanoma bone metastasis in mice. However, each alone did not show an anti-metastatic effect [143].

Although aspirin is a potent inactivator of cyclooxygenase-1 (COX-1), thus an inhibitor of platelet function, its failure to demonstrate an anti-metastatic role in some studies may be due to the ability of the tumour to activate platelets efficiently without COX-1-dependent synthesis of thromboxane A2 (TxA2), a hormone responsible for promoting platelet activation and aggregation. For example, limited effect of aspirin has been observed on platelet activation, aggregation and adhesion with agonists such as ADP, thrombin, high-dose collagen and elevated shear stress [145,146,147,148].

#### 8.1.2. Clinical Studies

The therapeutic use of aspirin has been extensively studied in colon cancer. In 1988, Kune and colleagues investigated the association of risk of colorectal cancer with medication use and found a statistically significant lower incidence of colorectal cancer cases among users of aspirin-containing medication [149]. In the subsequent APACC trial, 272 patients with a history of colorectal adenomas (an early sign of abnormal cell growth in the colon) were randomised to daily lysine acetylsalicylate (160 or 300 mg/day) or placebo for four years. The daily use of soluble aspirin showed a positive effect in reducing adenoma recurrence after one year of starting treatment as confirmed by colonoscopy [150]. In a larger double-blinded clinical trial involving 1121 patients with a recent history of adenomas, daily use of low-dose aspirin (81 mg) showed a moderate reduction in the incidence of one or more adenomas compared to placebo after one year [151]. A significant decrease in the size of polyps in patients with familial adenomatous polyposis was observed in the aspirin group compared to the placebo group in a separate randomised double blinded clinical trial [152]. In all the above studies, aspirin showed a positive effect, decreasing the very early stages of carcinogenesis. In addition to its protective effect, aspirin (81 to 325 mg once or more per day) use after diagnosis was associated with improved overall survival and decreased colorectal cancer-specific and overall mortality [153]. In the CAPP2 randomised controlled clinical trials in patients with Lynch syndrome (genetic mutations that increase the chance of developing cancer), regular use of aspirin (600 mg/day) also reduced the incidence of cancer [154]. Cao et al., have recently reported the results of a 32 years follow-up study which corroborated previous findings showing a positive effect of long-term aspirin use (81 to 325 mg at least two times a week) in reducing the incidence of cancer, especially gastrointestinal tumours [155]. Moreover, Frouws et al., have demonstrated that aspirin use (100 mg/day or lower) after diagnosis can significantly improve overall survival of gastrointestinal cancer [156]. Risch et al., have also documented a reduction in risk of pancreatic cancer by regular use of aspirin [157].

Since cancer and cardiovascular disorders are more prevalent in the elderly population, retrospective analysis of patient data pooled from large randomised clinical trials designed to examine daily aspirin in prevention of cardiovascular disease has been reviewed for an association between daily aspirin intake and incidence of cancer. In 2012, Rothwell and colleagues analysed data from five large randomised clinical trials of daily aspirin use (≥75 mg) including the UK Thrombosis Prevention Trial (TPT). In the TPT trial, aspirin was formulated as slow release to inhibit platelet function with minimal systemic bioavailability. In concordance with several animal studies, aspirin showed a reduction in cancer metastasis in the TPT trial consistent with a platelet-mediated effect [7]. Holmes and colleagues suggested a further reason for targeting platelets in cancer apart from it’s effect on metastasis; they demonstrated a role of aspirin in decreasing VEGF levels released from thrombin-activated platelets and associated tamoxifen use. Selective oestrogen receptor modulators like tamoxifen are extensively used in hormonal responsive breast cancer and are associated with increased plasma and platelet-derived VEGF [158].

In breast cancer, aspirin use (75 mg/day) after diagnosis reduced all-cause mortality and breast cancer-specific mortality in an observational study. This study included 4627 patients with 22% of females prescribed aspirin after diagnosis [159]. More convincing evidence came from a large prospective observational study including 4164 females diagnosed with breast cancer between 1976 and 2002, who were followed up until 2006. Aspirin use was associated with decreased distant recurrence and death either from breast cancer or any other cause [160]. A recent meta-analysis has reviewed the association between aspirin use and mortality in breast cancer and concluded that there is a small positive effect of aspirin in improving survival in breast cancer patients [161].

Contrary to these studies, Murray et al., reported little evidence of an association between low-dose aspirin intake (75 mg/day in 97.1% of the cohort) after diagnosis and cancer-specific death in a cohort of breast cancer patients in the UK [162]. Furthermore, Holmes et al., described a non-association between low-dose aspirin use (75 or 160 mg once or more per day) and low risk of breast cancer-related mortality in a nested case-control study in Sweden [163].The reason for these conflicting results may be the lack of data on patient acquiescence (compliance) and non-prescription use of aspirin. A phase II randomised clinical trial failed to show any positive effect of dual antiplatelet therapy (aspirin (325 mg/day) and clopidogrel (75 mg/day after 300 mg loading dose)) in reducing the number of circulating tumour cells (CTCs) in patients with metastatic breast cancer, the dual antiplatelet therapy was well tolerated with significant platelet inhibition after one month. Small sample size and a small number (less than five) of CTCs at baseline in the majority of the study population precluded a clear finding on the effect of antiplatelet therapy [164].

Head and neck cancer patients with high platelet counts have more than two times higher death rate compared to patients with mid-normal platelet counts. Antiplatelet (including aspirin) use was associated with a higher overall survival rate, with a more pronounced effect in the group of patients with high platelet count [165]. A retrospective study by Furlan et al., had a similar finding, and the authors suggested a synergetic effect of aspirin with radiotherapy [166].

Overall, several studies have documented the positive effect of aspirin (low or standard dose) on cancer incidence and cancer associated mortality in gastrointestinal tumours. However, it is not clear whether the effect of aspirin is related to its direct effect on cancer, platelets, both or unidentified mechanism. Therefore, randomised clinical trials are required to assess the use of aspirin and/or other antiplatelet medications in types of cancers associated with high risk of thrombosis e.g., pancreatic and ovarian cancer.

### 8.2. Other Antiplatelet Strategies in Cancer Therapy

While there has been considerable interest on the use of aspirin in cancer due to its direct effect on tumour cells and also its antiplatelet activity other antiplatelet drugs have also been examined in the context of cancer. More detailed information on general antiplatelet strategies can be found in reviews elsewhere [167,168]. Table 4 highlights studies that examined antiplatelet drugs in combination with chemotherapy in animal models or analysis of cancer incidence in patients taking antiplatelet for non-cancer diseases.

In addition to using antiplatelets in cancer treatment, their use may offer additional benefits in controlling venous thromboembolism (VTE) associated with cancer. Cancer patients have a high risk of developing VTE, which is related to low survival rate [169]. The cancer site and type are among factors that determine the incidence and severity of VTE in cancer [170]. For example, pancreatic cancer is associated with a high incidence of VTE [170,171,172]. Platelets are a key player in thrombosis, and several studies have shown a close interplay between pancreatic cancer cells and platelets [123,173]. Factors such as tumour cell induced platelet aggregation, and increased expression of pro-coagulant factors including tissue factor and thrombin, promote a pro-thrombotic state which ultimately contributes to the development of VTE [121]. Platelet count and activity may predict the risk of VTE in cancer patients [174,175]. The series of events that lead to cancer-associated VTE is still unclear; however recent studies indicate interactions between platelets, tumour and immune cells (especially neutrophils) can instigate the process. Neutrophil extracellular traps (NETs), generated from activated neutrophils, has been shown to act as a scaffold for platelet aggregation and thrombus formation [127,128]. Experimental evidence from infection and inflammation models suggests a role of activated platelets in promoting NET-derived thrombus formation [176,177,178]. Whether antiplatelet use may reduce the risk of NETs and VTE in cancer is still unknown.

## 9. Conclusions

The past 50 years have witnessed considerable advancement in our understanding of the tumour microenvironment and its role in cancer progression. Gasic et al. [6] provided the first experimental data for a pro-metastatic role of platelets in cancer. The accumulating evidence has since established the experimental rationale for targeting platelets in cancer.

Concomitant use of antiplatelet therapy in cancer patients carries both benefits and risks. Further collaboration between clinicians and research scientists is needed to investigate side effects and antiplatelet drug interactions with chemotherapeutic medications. The presence of specific cancer-related biomarkers could potentially predict patient response or necessity for antiplatelet therapy. For example, podoplanin is a potent platelet agonist and has been shown to be upregulated in several types of tumours. Aspirin is both an inhibitor of podoplanin-induced platelet aggregation in vitro and metastasis in vivo. Hence, podoplanin expression on tumour cells could serve as a predictive biomarker for individualised therapy.

Whether the addition of antiplatelet treatment alongside chemotherapeutic medication could increase therapeutic efficacy by reducing resistance, needs to be addressed. Nevertheless, the available evidence suggests targeting platelets in cancers known to have a high risk of thrombotic events, e.g., pancreatic and ovarian cancers, is therapeutically beneficial.

## Figures and Tables

**Table 1:** Normal physiological levels of angiogenic factors in platelets.

Angiogenic Factor	Normal Physiological Level in 10^6^ Platelets (Median (Range))
VEGF	0.68 (0.02–1.47) pg [75], 0.9 (0.1–2.3) pg [73]
PDGF	21 (12–33) pg [75], 19.1 (9.3–48.9) pg [73]
PF4	10 (2.4–22) ng [75], 10.2 (4.2–20.5) ng [73]
TSP-1	27 (7–54) ng [75]
bFGF	0.42 (0.15–0.75) pg [75]

**Table 2:** Commonly used anticancer drugs and risk of thrombocytopenia [94].

Drug	Side Effects
*Alkylating Agents*	
Cyclophosphamide	Lesser effect on peripheral platelet count compared to other alkylating agents.
Ifosfamide	Greater suppression of platelet count than cyclophosphamide
Carmustine	Delayed and prolonged suppression of platelet count, reaching a nadir at 4–6 weeks after administration, with slow reversal
Busulfan	Prolonged and cumulative effect lasting months or years
Thiotepa	Delayed effect compared to cyclophosphamide with platelet nadir at 3 weeks
Streptozocin	Suppression of platelet count in 20% of patients
Dacarbazine	Mild suppression of platelet count which is reversible within 1.2 weeks
Temozolomide	Similar to dacarbazine
Procarbazine	Suppression of platelet count after one week of initiating treatment and reversed within two weeks off treatment
*Platinum analogues*	
Cisplatin	Transient thrombocytopenia
*Antimetabolites*	
Methotrexate	Effect on platelets is completely reversed within two weeks. However, prolonged suppression may occur in patients with compromised renal function.
5-Florouracil	Thrombocytopenia, less often with infusion compared to bolus regimen
Cytarabine	Potent myelosuppression with severe thrombocytopenia
Gemcitabine	Mild haematological toxicities [95]. Myelosuppression is more prominent with longer duration infusion.
6-mercaptopurine	Gradual thrombocytopenia
Cladribine	Cumulative thrombocytopenia with repeated administration.
*Others*	
Topotecan	Neutropenia with or without thrombocytopenia.
Etoposide	Infrequent thrombocytopenia, which is usually, not severe.
Bleomycin	Minor myelosuppression
Mitomycin	Marked thrombocytopenia
Hydroxyurea	Occasional thrombocytopenia
Vorinostat	Thrombocytopenia is more prominent with intravenous administration.

**Table 3:** Summary of reported platelet effects on tumour growth.

Platelets Decrease Tumour Growth	Platelets Enhance Tumour Growth
In in vitro experiments, platelets showed a cytotoxic effect on cancer cells (Malme, a melanoma cell line, and 786, a renal carcinoma cwll line). The platelet effect was abrogated by aspirin [78]	Platelet-derived TGFβ1 enhances ovarian cancer growth in vitro and in vivo [84].
Platelets kill tumour cells (LU99A, a lung cell line, and K562, a chronic myeloid leukaemia cell line) via cyclooxygenase or nitric oxide-dependent pathways [79].	Platelets promoted proliferation of cancer cells (PLC/PRF/5, Hep3B and HepG2 cells hepatocellular carcinoma cell lines) in vitro via activation of the MAPK pathway [91].
Platelets kill tumour cells via activation of an apoptosis pathway in cancer cells (CEM, leukaemia cell line) through interaction between platelet-derived FAS-L and FAS receptor on the cancer cell [82].	Platelets enhance the growth of an SKOV3 human ovarian cancer xenograft [101].
Platelets prevented murine cancer cell growth (EG7 (H-2^b^), L1210, YAC-1 (H-2^a^) lymphoma cell lines, B16 H-2^b^, a melanoma cell line, and RM1 (H-2^b^), a prostate cancer cell line) by inducing cell cycle arrest rather than activating apoptosis [83].	Deposition of platelets in a solid tumour, as well as tumour growth (pancreatic islet insulinoma), was significantly reduced in P-selectin deficient mice [90].
	In a genetically modified lung cancer mouse model, PF4 enhanced platelet production and accumulation in the lung, which accelerated cancer progression [30].
	Platelets enhance the proliferation of colon and pancreatic cancer cells by upregulating the oncoprotein c-MYC [102].

**Table 4:** Examples of antiplatelet drugs investigated in the context of cancer.

Antiplatelet drugs	Study outcome	References
Dipyridamole and RA-233	In a pancreatic cancer mouse model, the combination of dipyridamole and RA-233 (cAMP-PDE inhibitor) reduced hepatic metastasis	[179]
Prasugrel	In the TRITON-TIMI 38 double-blinded randomised multicentre clinical trial of more than 13000 individuals assessing prasugrel versus clopidogrel in patients with acute coronary syndrome, prasugrel was associated with an increased incidence of gastrointestinal cancer. The exact mechanism is not entirely understood.	[180]
Clopidogrel	In prostate, breast and colorectal cancer patients, there was no increased risk of cancer-specific mortality among clopidogrel users. This study was in response to TRITON-TIMI 38 In a pancreatic cancer mouse model, clopidogrel reduced tumour growth, metastasis and thrombosis associated with cancer cell microparticle-derived tissue factor In a lung adenocarcinoma mouse model, clopidogrel reduced cancer growth and progression. [181]	[30,181,182]
Aspirin /Clopidogrel	In HBV transgenic mice, aspirin /clopidogrel delayed or prevented the development of hepatocellular carcinoma and improved the overall survival.	[183]
Clopidogrel with or without aspirin	In a large retrospective study involving 184,781 patients, use of clopidogrel with or without aspirin was associated with lower incidence of cancer	[184]
Ticagrelor	In melanoma and breast cancer mouse models, ticagrelor significantly reduced cancer metastasis and improved survival.	[185]
